# Supplementary material for: Two-dimensional electronic transport and surface electron accumulation in MoS2
Source: Nat Commun. 2018 Apr 12;9:1442. doi: 10.1038/s41467-018-03824-6 (PMC5897365; doi:10.1038/s41467-018-03824-6)
Supplement: Supplementary file 1 — Supplementary Information [file 41467_2018_3824_MOESM1_ESM.pdf]

Supplementary Information

Two-dimensional Electronic Transport and Surface Electron Accumulation in MoS_2

M. D. Siao[1], W. C. Shen[2], R. S. Chen[1,*], Z. W. Chang[3], M. C. Shih[4], Y. P. Chiu[3,4], C. -M. Cheng[5,6]

[1]Graduate Institute of Applied Science and Technology, National Taiwan University of Science and Technology, Taipei 10607, Taiwan

[2]Department of Electronic Engineering, National Taiwan University of Science and Technology, Taipei 10607, Taiwan

[3]Department of Physics, National Taiwan Normal University, Taipei 11677, Taiwan

[4]Department of Physics, National Taiwan University, Taipei 10617, Taiwan

[5]National Synchrotron Radiation Research Center, Hsinchu 30076, Taiwan

[6]Department of Physics, National Sun Yat-Sen University, Kaohsiung, 80424, Taiwan

[*]rsc@mail.ntust.edu.tw

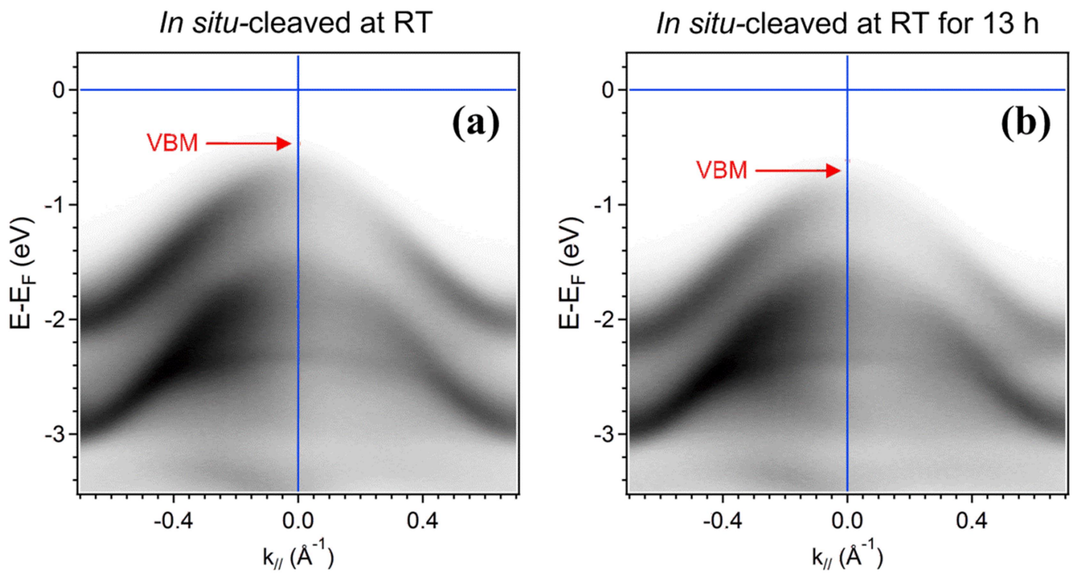

Supplementary Figure 1. Aging effect at room temperature (RT) of the *in situ*-cleaved fresh surfaces of a MoS_2 single crystal. The E versus $k_{\parallel}$ valance band measurements for the (**a**) *in situ*-cleaved surface at RT and (**b**) the *in situ*-cleaved surface at RT for 13 h of a MoS_2 crystal recorded with a 42-eV photon energy at RT. The observed overlap band structure can be attributed to a cleaved surface with two different crystal facets illuminated simultaneously by larger incident beam spot.

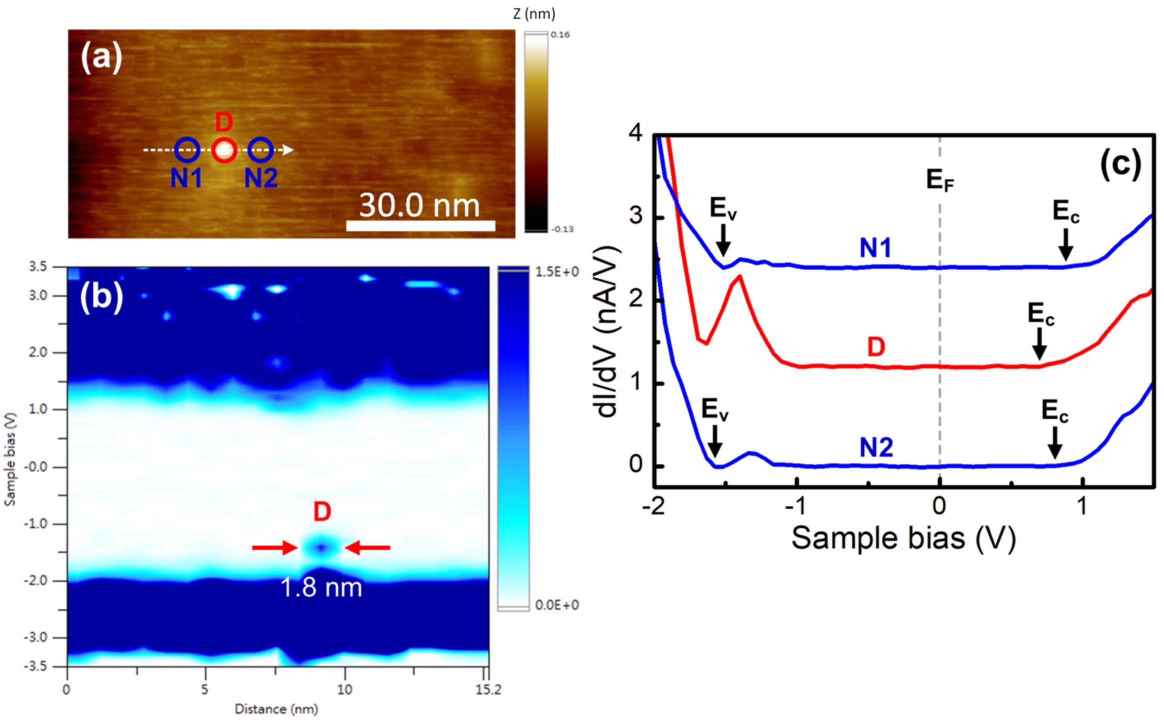

Supplementary Figure 2. STM/STS characterization of sulfur vacancies (SV) in the fresh MoS_2 surface. (**a**) STM image and (**b**) its STS mapping targeting an area with a point defect in the fresh MoS_2 surface. The white dash arrow in (a) indicates the scanning region of STS mapping. The red arrows in (b) indicate the influence region of the point defect labelled with D. (**c**) STS spectra measured at the defect center (position D) and its nearby area (positions N1 and N2) marked in (a).

Supplementary Note 1. By taking the STS spectra at the defect center (position D) and its nearby area (positions N1 and N2), we can clearly observe an extra peak close to the valance band (VB) edge centered at -1.4 V for the spectrum measured at position D as shown in Supplementary Figure 2(c). The similar feature is not so significant at the positions near the defect site (N1 and N2). According to the literatures, the presence of the SV introduces the defect states in the bandgap close to the VB edge.[1-4] The Fermi level at the defect center (D) exhibiting the blue-shift for 0.12–0.20 eV compared to those of the nearby positions (N1 and N2) indicates the *n*-type nature of the SV, which is also consistent with the previous reports.[5]

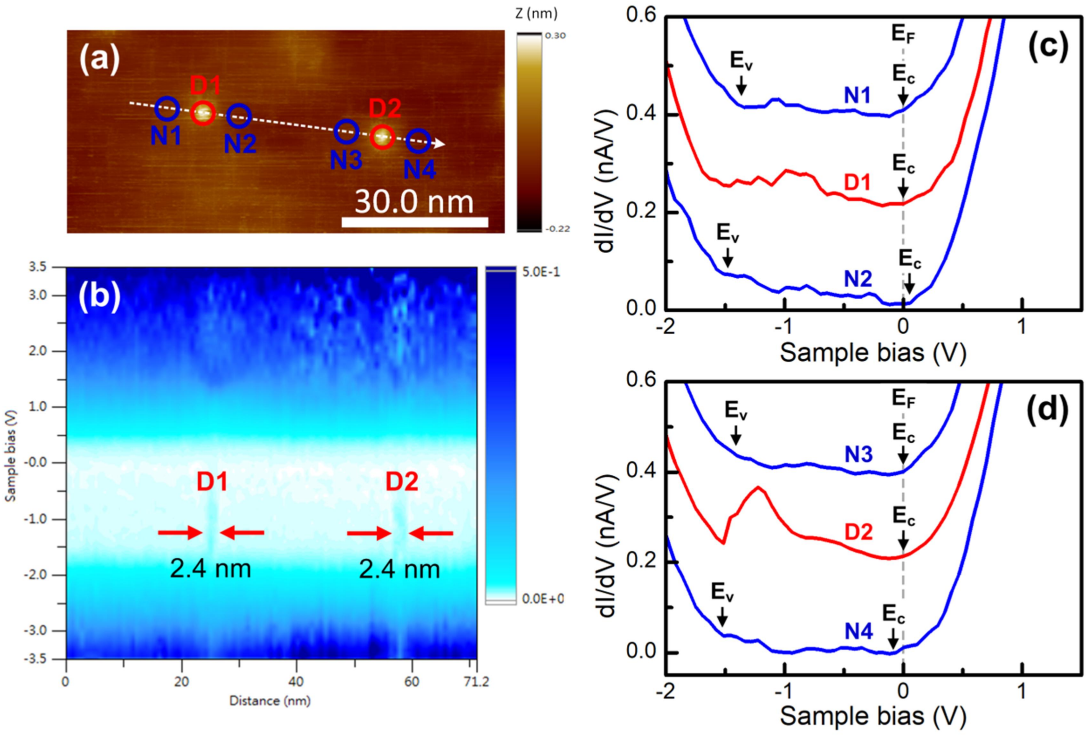

Supplementary Figure 3. STM/STS characterization of sulfur vacancies (SV) in the non-fresh MoS_2 surface. (**a**) STM image and (**b**) its STS mapping targeting an area with two point defects in the pristine (non-fresh) MoS_2 surface. The white dash arrow in (a) indicates the scanning region of STS mapping. The red arrows in (b) indicate the influence regions of the point defects labelled with D1 and D2. (**c**) STS spectra measured at the defect center (position D1) and its nearby area (positions N1 and N2) marked in (a). (**d**) STS spectra measured at the defect center (position D2) and its nearby area (positions N3 and N4) marked in (a).

Supplementary Note 2. According to the STS spectra in Supplementary Figures 3(c) and 3(d), we can also observe the similar features close to the valance band edge at the positions of defect centers (D1 and D2). The intensity of the extra peak in the non-fresh surface is not as high as that in the fresh sample, which is probably due to the long-term exposure in air. Santosh et al suggest that the oxygen adsorption could suppress the states originating from the SV.[4] The energy distribution and its density of states (DOS) could be slightly changed by the interaction of SV and oxygen molecules. The increase of the SV density could make a broader distribution of the defect states.[2] These statements probably can explain that the SV-related features are

slightly different in energy position and relative intensity in the STS spectra shown in Supplementary Figures S2(c), S3(c) and S3(d).

Supplementary Note 3. Calculation of surface electron diffusion length in MoS_2.

The surface electron accumulation originates from the electron injection from the surface (donor-like surface states) into the bulk. The surface-bulk interface is somewhat similar to the n^{++}-n junction. To understand to what thickness the electron accumulation persists, we can estimate the diffusion length of electron (L_n) in the intrinsically n-type MoS_2. According to the one-dimensional continuity equation at steady state: $D_n \frac{d^2(\delta n)}{dx^2} - \frac{\delta n}{\tau_n}$, the excess electron concentration (δn) as a function of space (x) is written as $\delta n(x) = \delta n(0)\exp(-x/L_n)$, where $\delta n(0)$ is the excess electron concentration at $x = 0$ and $L_n = (D_n \tau_n)^{1/2}$, where D_n is the diffusion coefficient of electron and τ_n (~1 ns)[6] is the electron lifetime.[7] The D_n of MoS_2 can be calculated by the Einstein equation, $D_n/\mu_n = kT/q$, where μ_n is the electron mobility, k is Boltzmann's constant, T is the temperature set at 300 K, and q is the elementary charge.

The layer material like MoS_2 has strong anisotropic transport properties. The conductivity and effective mass of electron perpendicular to the c-axis (in-plane) is approximately three orders of magnitude higher that that along c-axis (out-of-plane).[8] According to the references, the in-plane mobility values of MoS_2 bulk[9,10] are 32–100 $cm^2V^{-1}s^{-1}$ and so the out-of-plane mobility values are inferred at the range of 0.032–0.1 $cm^2V^{-1}s^{-1}$. Because the electron injection is along the c-axis, the out-of-plane mobilities were adopted for the D_n calculation. The estimated D_n are 8.3×10^{-4}–2.6×10^{-3} cm^2s^{-1} and the obtained L_n locates in the range of 9.1–16 nm.

Supplementary References

1. Fuhr, J. D., Saul, A., Sofo, J. O. Scanning tunneling microscopy chemical signature of point defects on the MoS_2(0001) surface. *Phys. Rev. Lett.* **92**, 026802 (4 pages) (2004).
2. Qiu, H. *et al*. Hopping transport through defect-induced localized states in molybdenum disulphide. *Nature Commun.* **4**, 2642 (6 pages) (2013).
3. Santosh, K. C., Longo, R. C., Addou, R., Wallace, R. M., Cho, K. Impact of intrinsic atomic defects on the electronic structure of MoS_2 monolayers. *Nanotechnology* **25**, 375703 (6 pages) (2014).
4. Akdim, B., Pachter, R., Mou S. Theoretical analysis of the combined effects of sulfur vacancies and analyte adsorption on the electronic properties of single-layer MoS_2. *Nanotechnology* **27**, 185701 (10 pages) (2016).
5. McDonnell, S., Addou, R., Buie, C., Wallace, R. M., Hinkle, C. L. Defect-dominated doping and contact resistance in MoS_2. *ACS Nano* **8**, 2880-2888 (2014).
6. Wang, H., Zhang, C., Rana, F. Surface Recombination Limited Lifetimes of Photoexcited Carriers in Few-Layer Transition Metal Dichalcogenide MoS_2. *Nano Lett.* **15**, 8204-8210 (2015).
7. Neamen, D. A. *Semiconductor Physics and Devices: Basic Principles*, 3rd edition (McGraw-Hill Inc., New York, **2003**), Chap. 6, pp. 206-207.
8. Guha Thakurta, S. R., Dutta, A. K. Electrical conductivity, thermoelectric power and Hall effect in *p*-type molybdenite (MoS_2) crystal. *J. Phys. Chem. Solids* **44**, 407-416 (1983).
9. Tiong, K. K., Liao, P. C., Ho, C. H., Huang, Y. S. Growth and characterization of rhenium-doped MoS_2 single crystals. *J. Crystal Growth* **205**, 543-547 (1999).
10. Fivaz, R., Mooser, E. Mobility of charge carriers in semiconducting layer structures. *Phys. Rev.* **163**, 743-755 (1967).
